# Intensive care management of patients with COVID-19: a practical approach

**DOI:** 10.1186/s13613-021-00820-w

**Published:** 2021-02-18

**Authors:** Ludhmila Abrahão Hajjar, Isabela Bispo Santos da Silva Costa, Stephanie Itala Rizk, Bruno Biselli, Brenno Rizerio Gomes, Cristina Salvadori Bittar, Gisele Queiroz de Oliveira, Juliano Pinheiro de Almeida, Mariana Vieira de Oliveira Bello, Cibele Garzillo, Alcino Costa Leme, Moizo Elena, Fernando Val, Marcela de Almeida Lopes, Marcus Vinícius Guimarães Lacerda, José Antonio Franchini Ramires, Roberto Kalil Filho, Jean-Louis Teboul, Giovanni Landoni

**Affiliations:** 1 Instituto Do Coração, University of São Paulo Medical School, Av. Dr. Enéas de Carvalho Aguiar, 44, São Paulo, SP Brazil; 2 Instituto Do Câncer, Universidade de São Paulo, São Paulo, Brazil; 3 Hospital Sírio Libanês, São Paulo, SP Brazil; 4 IRCCS San Raffaele Scientific Institute, Milan, Italy; 5 Instituto Leônidas & Maria Deane, Fiocruz, Manaus, Brazil; 6 Hospital da Cidade, Salvador, Bahia Brazil; 7 Fundação de Medicina Tropical Dr. Heitor Vieira Dourado, Manaus, Brazil; 8 Medical Intensive Care Unit, Bicêtre Hospital, Paris-Sud University Hospitals, Le Kremlin Bicêtre, France; 9 Vita-Salute San Raffaele University, Milan, Italy

**Keywords:** COVID-19, Intensive care unit, Circulatory support and invasive ventilation

## Abstract

SARS-CoV-2, the causative agent of coronavirus disease 2019 (COVID-19), is responsible for the largest pandemic facing humanity since the Spanish flu pandemic in the early twentieth century. Since there is no specific antiviral treatment, optimized support is the most relevant factor in the patient's prognosis. In the hospital setting, the identification of high-risk patients for clinical deterioration is essential to ensure access to intensive treatment of severe conditions in a timely manner. The initial management of hypoxemia includes conventional oxygen therapy, high-flow nasal canula oxygen, and non-invasive ventilation. For patients requiring invasive mechanical ventilation, lung-protective ventilation with low tidal volumes and plateau pressure is recommended. Cardiovascular complications are frequent and include myocardial injury, thrombotic events, myocarditis, and cardiogenic shock. Acute renal failure is a common complication and is a marker of poor prognosis, with significant impact in costs and resources allocation. Regarding promising therapies for COVID-19, the most promising drugs until now are remdesivir and corticosteroids although further studies may be needed to confirm their effectiveness. Other therapies such as, tocilizumab, anakinra, other anti-cytokine drugs, and heparin are being tested in clinical trials. Thousands of physicians are living a scenario that none of us have ever seen: demand for hospital exceed capacity in most countries. Until now, the certainty we have is that we should try to decrease the number of infected patients and that an optimized critical care support is the best strategy to improve patient’s survival.

## Introduction

Since December 31, 2019, when China reported a series of cases of acute respiratory failure caused by a new species of coronavirus, SARS-CoV-2, more than 50 million new cases and almost 1,260,000 deaths have been confirmed worldwide. In Brazil, 5,664,115 cases were reported with 162,397 deaths from the disease by November 8th, 2020 [1]. Its rapid spread and high lethality, especially in the most fragile groups such as the elderly and those with comorbidities, make this pandemic a new challenge faced by modern medicine.

The pathophysiology of COVID-19 is complex, and the disease may compromise lung, heart, brain, liver, kidney, and of the coagulation system. COVID-19 can result in myocarditis, cardiomyopathy, ventricular arrhythmias, acute coronary syndrome, and shock [2–8]. Venous and arterial thromboembolic events occur in 31–59% of hospitalized patients with COVID-19 [5, 6].

This publication aims to provide a specialist consensus on specific management of COVID-19 in intensive care, covering from the admission criteria in the intensive care units (ICU) to antiviral treatment, with sections on ventilatory, hemodynamic, and metabolic support. We searched PubMed, Medrxiv, and Embase using the search terms coronavirus, COVID-19, SARS-CoV-2, severe acute respiratory syndrome COVID-19, critically ill, and intensive care unit for studies published from December 31, 2019, to June 11st, 2020, and selected manually the relevant articles. We selected articles relevant to a general medicine readership, prioritizing randomized clinical trials, systematic reviews, and clinical practice guidelines.

## ICU admission criteria for adult patients infected with COVID-19

Authorities from the Chinese Center for Disease Control and Prevention reported that, among more than 44,000 confirmed cases of COVID-19, about 81% were asymptomatic or presented mild symptoms such as cough, fever, fatigue, and myalgia [9]. Although for these cases, home management and self-isolation are the appropriate measures, 14% developed a severe form of the disease and 5% were critical, requiring hospitalization and ICU admission, respectively [9]. Severe patients with COVID-19 usually present respiratory rates ≥ 30 breaths per minute, oxygen saturation ≤ 93%, and lung infiltrates > 50% [9], and are at high risk for clinical deterioration and for developing critical illness, including acute respiratory distress syndrome (ARDS) [10]. Hospitalization should be warranted for patients who develop severe symptoms; however, ICU admission has been reserved for the most severe forms, depending on the capacity of the health care system. Despite differences in culture and practices around the world, most centers report that around 25% of hospitalized patients require ICU admission [11, 12].

Patients with the severe form of the disease must be closely monitored, since rapidly progression from moderate to severe ARDS may occur. Acute hypoxemic respiratory failure is the most common complication occurring in 60–70% of patients admitted to the ICU [11]. Patients at high risk for ARDS development are those older than 65 years old, presenting high fever (T > 39ºC), neutrophilia, lymphocytopenia, elevated markers of hepatic and renal failure (aspartate aminotransferase, alanine aminotransferase, creatinine, and urea), elevated acute-phase proteins as markers of inflammation (high-sensitivity C-reactive protein, procalcitonin, and serum ferritin), and elevated coagulation function-related indicators (prothrombin time, fibrinogen, and D-dimer) [1, 10].

Admission criteria include oxygen requirements equal or superior to 6–8 l/min to reach a peripheral oxygen saturation ≥ 90–92%, respiratory failure, shock, acute organ dysfunction, and patients at high risk for clinical deterioration. However, in many countries, due to the shortage of ICU beds, usually only patients requiring intubation and invasive mechanical ventilation were admitted to ICU.

## Pulmonary impairment, physiopathology, and ventilation strategies

**Fig. 1:**
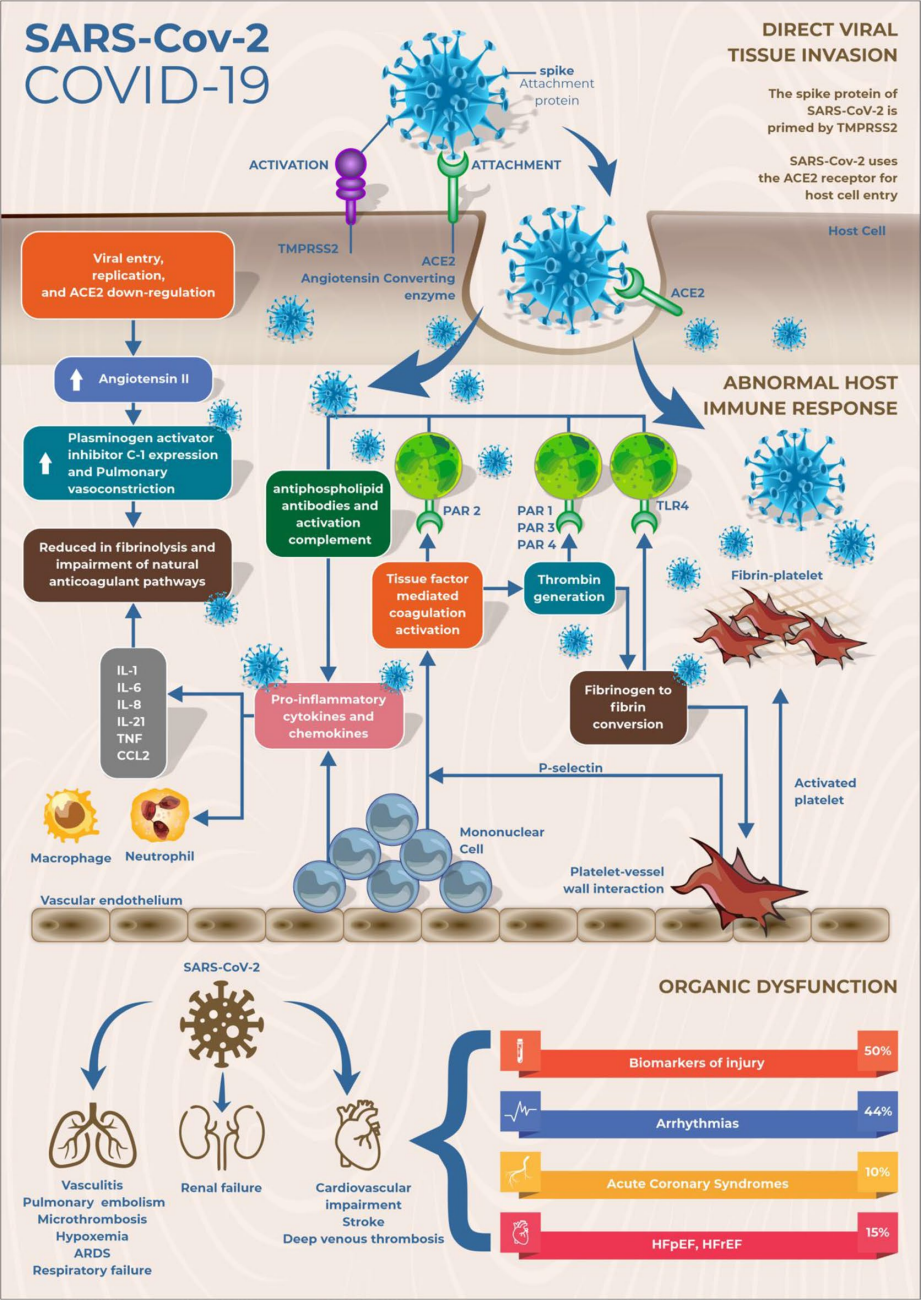
The pathophysiology of SARS-CoV-2 infection. SARS-CoV-2, via its surface spike protein, binds to the human ACE2 receptor after spike protein activation by TMPRSS2. This results in down-regulation of ACE2 and increased angiotensin II levels and consequently increased plasminogen activator inhibitor C-1 expression and reduced fibrinolysis. The disease it causes is associated with an increase in inflammatory cytokines and coagulation disorders, with predisposition to thrombus formation. Mononuclear cells interact with activated platelets and the coagulation cascade, which activate 1 inflammatory cells by binding thrombin and tissue factor with specific protease activated receptors and by binding fibrin to Toll-like receptor 4. The activation of inflammatory cells results in the release of pro-inflammatory cytokines, leading to impairment of the natural coagulation pathways and shut down of fibrinolysis. This state of hyper inflammation and hypercoagulability leads to multiple organ dysfunction, most commonly affecting the lungs, heart and kidneys. *ACE2* angiotensin-converting enzyme-2, *aPTT* activated partial thromboplastin time, *ARDS* acute respiratory distress syndrome, *COVID-19* coronavirus disease 2019, *HFpEF* heart failure preserved ejection fraction, *HFrEF, *heart failure reduced ejection fraction, *IL* interleukin, *PAR* protease-activated receptor, *PT *prothrombin time, *SARS-COV-2*severe acute respiratory syndrome coronavirus 2, *TMPRSS2* transmembrane protease serine, *TLR4* Toll-like receptor 4, *TNFα* tumor necrosis factor-α

The pathophysiology of COVID-19-induced ARDS involves characteristic properties which make it different from other causes of ARDS: patients present an intense endothelial dysfunction with a thromboinflammatory state. Multiple mechanisms of dysregulation in the pulmonary perfusion exist in COVID-19: the abolition of hypoxic pulmonary vasoconstriction, excessive pulmonary vasoconstriction; and microthrombosis or macrothrombosis, leading to increased dead space [13, 14]. Pulmonary microthrombosis and endothelial damage that result in V/Q (ventilation/perfusion) mismatch, hypoxemia, and vasodilation (Fig. 1) [15–17]. Increased inflammatory and thrombotic biomarkers are associated with severe clinical presentation and mortality in COVID-19 patients. High levels of D-dimer, IL-6, C-reactive protein, procalcitonin, troponin, LDH, and ferritin are detected in severely ill patients [18].

Hospitalization trends vary with age and may reach around 20% of COVID-19 patients [19]. In hospitalized patients, ventilatory support may vary from the need for O_2_ supplementation through a nasal catheter to invasive mechanical ventilation or extracorporeal membrane oxygenation (venovenous ECMO) in patients with the most severe forms of ARDS. In general, patients must be maintained with the minimum amount of supplemental O_2_ for a SpO_2_ between 92 and 96%. Without BGA, the degree of hypoxemia can be estimated by SpO_2_/FiO_2_ ratios, with values ≤ 315 suggesting ARDS [20].

Non-invasive ventilation: Health services have been and are predicted to be overloaded in most large centers as a result of the spreading COVID-19 pandemic, leading mainly to the lack of ICU beds and insufficient number of mechanical ventilators for patients who need them [21]. Many hospitals have adopted non-invasive ventilation (NIV) as an attempt to prevent respiratory failure from evolving into severe forms that require invasive ventilatory support.

Both the European Society for Intensive Care Medicine (ESICM) with the international Surviving Sepsis Campaign: Guidelines on the management of critically ill adults with coronavirus disease 2019 (COVID-19), and the National Health Service of England (NHS-England) recommend the use of NIV as an initial measure for respiratory failure in patients with COVID-19 (weak recommendation, low-quality evidence) [22, 23]. It is still an ongoing debate about which would be the most recommended non-invasive interface and technique: NIV devices such as BIPAP, or the use of high-flow nasal cannulas (HFNC).

The prone position in non-intubated patients were tested in some initial studies [24–26]. Patients who tolerated more than 3 h in prone position present a substantially improve in oxygenation from supine to prone positioning. However, only about half of the patients maintain the benefit after resupination [25, 26]. Further studies are needed to reinforce the real benefit of this intervention.

### Invasive mechanical ventilation

ARDS is defined as a form of inflammatory pulmonary edema of non-cardiogenic etiology, with a reduction in the areas of normoventilated lung and consequent reduction in respiratory compliance and shunt effect. The Berlin definition proposed categories of ARDS based on degree of hypoxemia: mild (200 mmHg < PaO_2_/FIO_2_ ≤ 300 mmHg), moderate (100 mmHg < PaO_2_/FIO_2_ ≤ 200 mmHg), and severe (PaO_2_/FIO_2_ ≤ 100 mmHg) and variables for severe ARDS: radiographic severity, respiratory system compliance (≤ 40 mL/cm H_2_O), positive end-expiratory pressure (≥ 10 cm H_2_O), and corrected expired volume per minute (≥ 10 L/min) [27].

To manage these patients, maneuvers that lead to recruitment of collapsed areas are usually applied, such as increased positive end-expiratory pressure (PEEP), alveolar recruitment maneuvers, and prone position, leading to a reduction in elastance and increased compliance [28]. Prone positioning presents the potential benefit of a relieve of severe hypoxemia due to reduction of overinflated lung areas, promoting alveolar recruitment and decreasing ventilation/perfusion mismatch. This intervention might be considered in patients with PO2/FiO2 < 150, in the absence of contraindications [29, 30]. The main objective of mechanical ventilation in these patients is to maintain a lung-protective strategy for all patients with ARDS, defined as targeting a tidal volume of 4 to 8 mL/kg predicted body weight (PBW) and a plateau pressure of less than 30 cmH_2_O [28].

A group of experts hypothesize that in COVID, there may be two phenotypes of ARDS [21]. Patients often exhibit normal compliance even in the presence of severe hypoxemia, with normal or even increased minute ventilation, and more than half of these patients do not appear dyspneic. Radiologically, such patients have ground-glass tomographic lesions indicative of interstitial and non-alveolar edema, and these infiltrates are relatively limited in extent at this stage. These patients are called “type L” (“low elastance”), with additional main characteristics of high compliance, low response to PEEP, and low lung weight estimated by chest computed tomography (CT) [21]. Patients may evolve with progressive clinical improvement or, whether due to individual predisposing factors or inadequate management, evolve with a more severe form closer to the classic ARDS. This is named as “type H” (from “high elastance”), showing also low compliance, high response to PEEP, and high lung weight estimated on chest CT [31]. It should be highlighted that this division is conceptual, to facilitate the understanding of the respiratory condition, with types “H” and “L” representing the ends of a spectrum that frequently overlap [31].

### Mechanical ventilation strategy according to patient phenotypes (“type L” or “type H”)

In severe cases of respiratory failure, as frequently seen in SARS-CoV-2-related ARDS, severe hypoxemia can lead to a persistent increase in respiratory effort, with consequent self-induced lung injury (P-SILI). In addition, other factors such as fluid overload or SARS-1 CoV-2-induced myocardial injury may also play important roles in worsening of the condition through pulmonary congestion [32]. Thus, a mechanical ventilation strategy must take into account the multiple mechanisms of lung injury and the different presentations of the disease—conventional form of ventilation in ARDS will not always be the most appropriate, as described below [31].

- Type L: it is suggested to ventilate “type L” patients, typically patients with good lung compliance, higher tidal volumes (VT) (around 7–8 mL/kg of ideal body weight). Higher VT helps to avoid reabsorption atelectasis and hypercapnia due to limited VT-induced hypoventilation. The rationale behind this strategy is as follows: the initial feature of these patients is the vasoregulation defect in the pulmonary capillaries—the reflex vasoconstriction that normally occurs in response to hypoxemia is not found in these patients due to endothelial changes and microthrombosis. Elevation of FiO_2_ may be sufficient in most patients not experiencing excessive respiratory effort, with maintenance of NIV with BIPAP or HFNC leading to slow and progressive improvement of hypoxemia and reversal of ARDS. However, if the inflammatory condition progresses, or if the patient's ventilatory effort is excessive, secondary pulmonary tissue stress may lead to P-SILI, with severe deterioration of lung function. At this point, intubation with adequate sedation/paralysis can interrupt the vicious cycle. These patients should be ventilated with lower PEEP (between 8 and 10 cmH_2_O) to avoid redirection of blood flow away from the aerated pulmonary capillaries, which would increase the shunt effect. As capillary hypoperfusion can also suffer a gravity-dependent effect, the prone position could be used as a strategy to minimize it and increase oxygenation.
- Type H: with disease progression and worsening of inflammatory edema, the patient may progress to “type H”. The pathophysiology of this progression is probably the result of a combination of factors: in addition to self-induced lesion (P-SILI), the viral lesion itself leads to uncontrolled inflammation and edema, with local and generalized thrombogenesis, intense release of cytokines, and right ventricular overload. The resulting pulmonary edema is close to classic ARDS presentation, with collapsed alveoli and extensive normoperfused and hypoaerated areas. In these more advanced cases, a mechanical ventilation strategy should be more traditional: elevated PEEP, VT < 6 mL/kg, driving pressure < 14 cmH_2_O, prone position, and alveolar recruitment maneuvers in refractory cases.

**Fig. 2:**
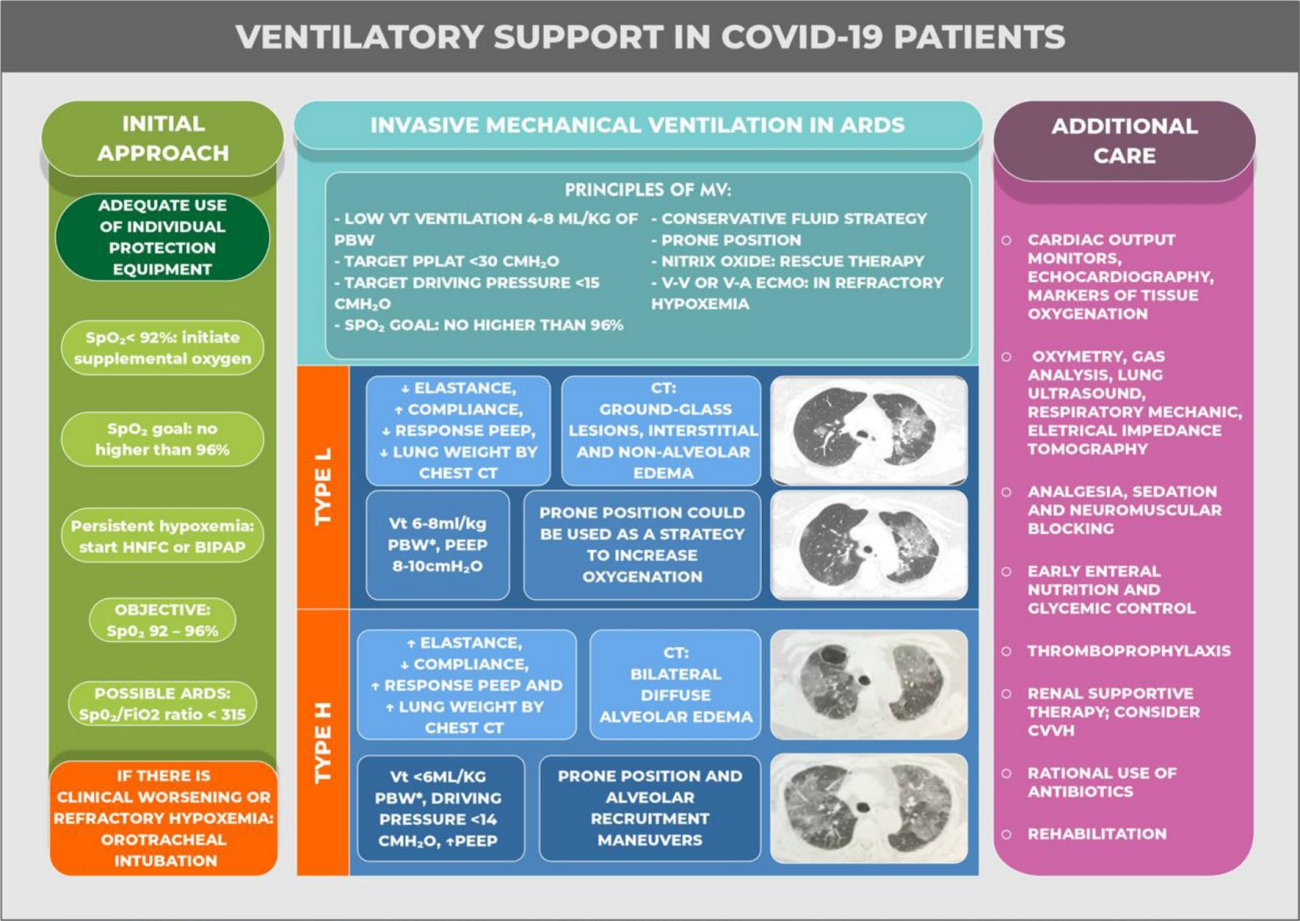
Ventilatory support in COVID-19 patients. *PBW* predicted body weight, *ARDS* acute respiratory distress syndrome, *BNP* brain natriuretic peptide, *CPAP* continuous positive airways pressure, *CT* computerized tomography, *CVVH* continuous venovenous hemofiltration, *CRP* C-reactive protein, *ECMO* extracorporeal membrane oxygenation, *LDH* lactic dehydrogenase, *PEEP* positive end-expiratory pressure, *PPlat* plateau pressure, *HNFC* high-flow nasal cannulas, *NIV* non-invasive ventilation, *PEEP* positive end-expiratory pressure, *SpO2* peripheral O2 saturation, *PaO2* partial pressure of oxygen, *Vt* tidal volume, *V-V* venovenous, *V-A* venous-arterial

As previously stated, categorization in two different profiles facilitates clinical management by indicating the need for different ventilatory approaches. However, due to the frequent overlap of the two types, individualization of ventilatory management is essential. In either case, patients with COVID-19 who undergo mechanical ventilation have an average recovery time of 1–3 weeks [33, 34]. The progress toward improvement is characteristically slow; therefore, prolonged sedation is often unavoidable. In most severe cases of ARDS and also in cases of non-protective ventilation or in the occurrence of asynchrony, neuromuscular blockage is useful, and complications such as polyneuropathy of the critically ill patient are usually diagnosed. Figure 2 shows the main principles of the management of COVID-19 respiratory failure.

## Cardiovascular impairment, physiopathology, and treatment strategies

**Fig. 3:**
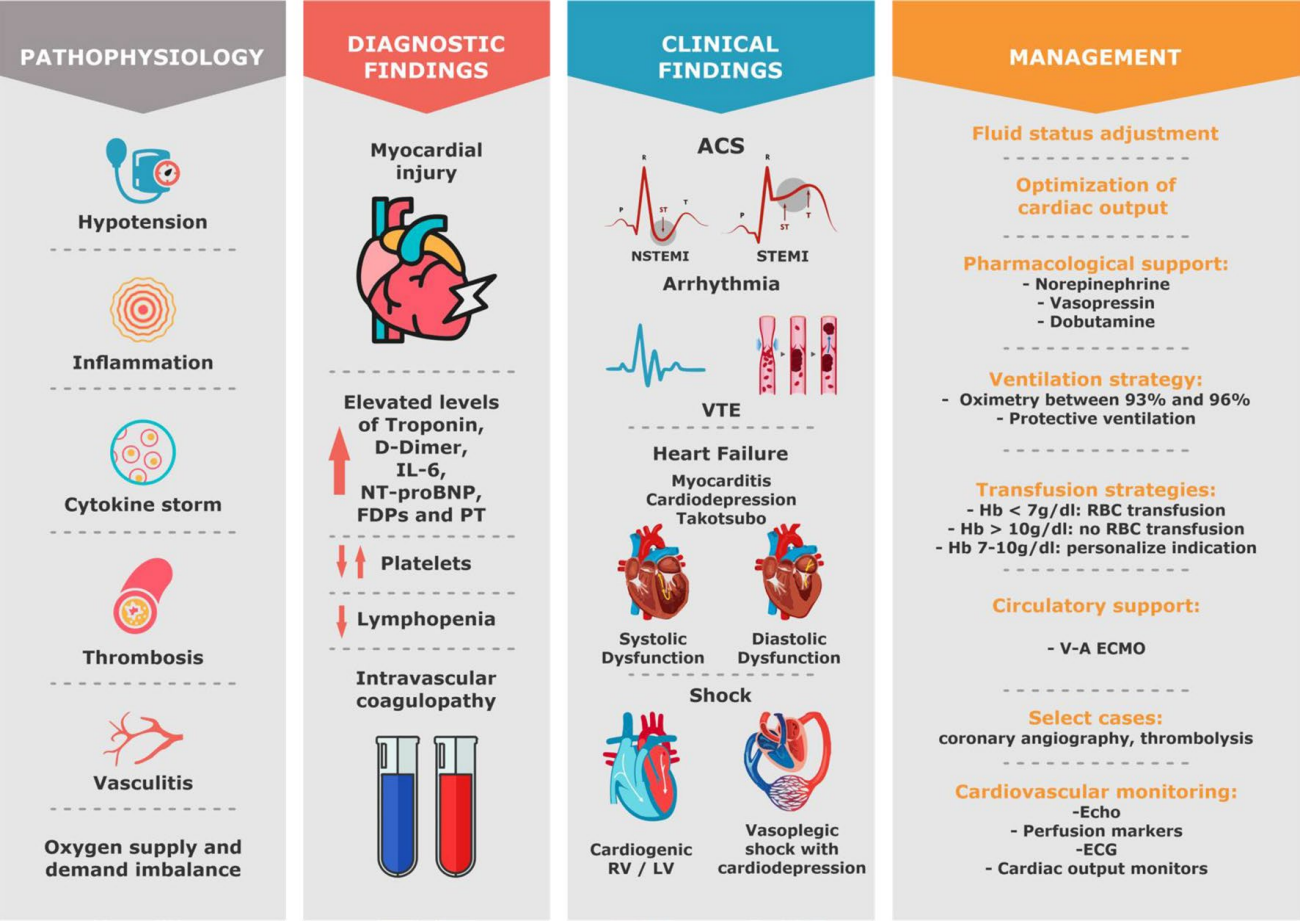
Cardiovascular involvement in patients with COVID-19; pathophysiology, diagnostic findings, most common clinical findings and proposed management. *IL-6* interleukine-6, *FDPs* fibrin degradation product, *PT* prothrombin time, *ACS* acute coronary syndrome, *NSTEMI* non-ST elevation myocardial infarction, *STEMI* ST elevation myocardial infarction, *VTE* venous thromboembolism, *RV* right ventricle, *LV* left ventricle, *Hb* hemoglobin, *V-A* venous-arterial, *Echo* echocardiography, *ECG* electrocardiogram

Cardiac injury is a common occurrence in patients with COVID-19. Multiple mechanisms are involved: virus direct toxicity, inflammation, thrombogenesis, endothelial injury, sympathetic overstimulation, myocarditis, hypoxemia, vasoconstriction, supply/demand disorder, low cardiovascular and respiratory reserve, and secondary infections. Different phenotypes result from this: cardiac injury alone, myocarditis with heart failure, arrhythmias, venous and arterial thromboembolism, acute coronary syndrome, and shock (Fig. 3) [4, 35, 36].

A recent study that included 100 patients with COVID-19 submitted to echocardiography within 24 h of admission showed that the most common findings were right-ventricular (RV) dilatation and dysfunction (39% of patients), followed by left-ventricular (LV) diastolic dysfunction (16%) and LV systolic dysfunction (10%) [37]. In hospitalized patients with confirmed COVID-19, the incidence of shock is 20–35% [38, 39]. Among patients 1 who received IMV, about 95% may need vasopressor support [40].

The main goal of shock and hemodynamic instability management of patients with COVID-19 severe illness is to restore arterial pressure and to optimize cardiac output with the ultimate goal to improve or preserve organ perfusion. Severe lung injury and mechanical ventilation also contribute to deleterious hemodynamic effects. The extensive viral infection and ARDS induced by COVID-19 results in diffuse lung inflammation, consolidation, marked microvascular thrombosis, endothelial dysfunction, and vasoconstriction [41]. In addition, hypoxemia and dead space lead to a rise in pulmonary vascular pressure and resistance and right-ventricular (RV) afterload increase [42].

The increase in RV afterload and preload may result in RV dilatation, in a septal shift toward the left ventricle (LV) and then in a decrease in LV filling that eventually result in low cardiac output and hemodynamic deterioration [42].

Acute Cor Pulmonale (ACP) is a relatively common occurrence in severe COVID-19 patients. In some patients, ACP can be the result of acute pulmonary embolism, a frequent event in the course of COVID-19 [43], which should prompt a specific therapy [44].

A conservative fluid strategy is usually recommended in patients with ARDS. Aggressive fluid administration and hypervolemia is associated with longer ICU stay, prolonged ventilator dependence, and higher mortality [22, 42, 45, 46]. However, hypovolemia can occur in COVID-19, especially in the early phase, or even in later phases due to intense associated sepsis (increased capillary leakage and increased venous capacitance). Uncorrected hypovolemia may lead to peripheral organ hypoperfusion, facilitate thrombi formation in the context of severe COVID-19 coagulopathy, and might even aggravate hypoxemia due to a low PvO_2_ effect in case of high degree of pulmonary shunt fraction. It is thus important to assess the benefit/risk ratio of fluid administration. The expected benefit could be assessed by performance of fluid responsiveness tests, administering bolus of hypotonic crystalloids (lactated`s Ringer), and evaluating dynamic variables such as cardiac index and velocity–time integral [47]. Use of tidal volume challenge assessing the changes in pulse pressure variation during a transient increase in tidal volume (e.g., from 6 to 8 mL/kg) [48] is an excellent option in COVID-19 ARDS patients often deeply sedated. If available, advanced hemodynamic monitoring technologies 1 such as transpulmonary thermodilution may help assessing the fluid infusion benefit/risk ratio.

Additionally, COVID-19 myocardial depression may develop at any phase of the disease, sometimes with fulminant myocarditis which might occur in about 1% of hospitalized patients [49, 50]. Early detection of myocardial involvement through the measurement of troponin and natriuretic peptide concentrations and echocardiography is recommended [4, 11]. A recent paper showed that even in less severe forms of disease, myocardial inflammation may persist in almost 60% of patients 70 days after disease [51]. Point-of-care echocardiography might help in the diagnosis of shock and in the non-invasive assessment of preload responsiveness [47, 52–54].

Norepinephrine is the first-line vasopressor in patients with hemodynamic instability and COVID-19 [22, 45, 55]. In patients with ARDS, norepinephrine also can improve RV function by restoring mean arterial pressure and thus RV blood supply [42]. If norepinephrine is unable to achieve adequate mean arterial pressure, vasopressin could be added as a second-line vasoactive agent to reach the target blood pressure [22]. Vasopressin might be used as first vasopressor, mainly in cases of atrial fibrillation, pulmonary hypertension, and acute renal failure [56]. Angiotensin 2 (Ang-2) has also been successfully used in COVID-19 patients in a few cases [57]. Although there are no definite trials to support Ang-2′s superiority over conventional vasopressors in COVID patients with vasodilatory shock, there is a physiologic rationale for using the drug. Dobutamine is the inotropic agent the most used in case of acute heart failure. It is indicated in the presence of cardiac dysfunction and in the occurrence of tissue hypoxia after fluid status adjustment and norepinephrine administration.

Prone positioning and inhaled selective pulmonary vasodilators have been used for patients with refractory hypoxemia; moreover, they may have a beneficial hemodynamic effect in particular by decreasing RV afterload and restoring RV function [58, 59]. Finally, in cases of refractory ARDS combined or not with refractory cardiogenic shock requiring high vasoactive doses, venovenous or veno-arterial ECMO might be considered to restore blood oxygenation and provide hemodynamic stability [60]. Although many new centers have been created and ECMO services are now available worldwide, access to ECMO is restricted, especially in low-income countries. Establishing models of care, in which severely ill patients who are eligible for ECMO are evaluated by systems that can be used to rapidly transfer and group high numbers of critically ill patients would be important to provide high-quality ECMO care during the pandemics. Another issue of interest in ECMO management is that the SARS-CoV-2-induced infection may be associated with higher rates of thrombotic events of the extracorporeal system during V-V ECMO therapy [61].

## Renal impairment, physiopathology, and treatment strategies

Although respiratory failure is the main dysfunction caused by SARS-CoV-2, other organs can also be affected, with cardiac and renal failures as the most relevant disorders [62]. Acute kidney injury (AKI) was present in 6.7% with a mortality rate as high as 91.7% in SARS-CoV (causative agent of SARS) [63]. Among 99 ICU COVID-19 patients, Fominsky et al. found that 72 (75.0%) developed AKI and 17 (17.7%) received continuous renal replacement therapy (CRRT) [64].

In a prospective cohort study that included 701 patients diagnosed with COVID-19, the assessment of renal function at admission showed that serum creatinine was elevated in 14.4% and urea in 13.1% of patients [65]. Abnormalities in the coagulation pathway, including prolonged partial thromboplastin time and high D-dimer, were more common in patients with elevated baseline serum creatinine. The risk factors associated with mortality were: proteinuria in any degree, hematuria, elevated basal creatinine, and renal failure AKIN (Acute Kidney Injury Net) 2 or more. There was a correlation between severity of kidney injury (AKIN stages) and death, with a fourfold higher risk of mortality among those with stage 3 AKIN [65].

### COVID-19 nephropathy

Findings like proteinuria and hematuria can occur after COVID-19 infection, with some individuals showing signs and symptoms of AKI. It has been demonstrated that RNA viruses are present in urine and renal tissue, indicating that the kidney may also be a target of COVID-19 infection 1 through direct viral invasion in the tubules and renal interstitium [66, 67].

Renal histopathology was examined in a series of autopsies of 26 patients who died of respiratory failure secondary to COVID-19. All patients had evidence of acute tubular injury of varying severity, and a number of other histopathological findings including clusters of erythrocytes and hemosiderin pigments were also present. Of the nine samples tested for the intracellular virus, coronavirus-like particles were identified in seven [66].

Renal failure due to COVID-19 has a multifactorial etiology, with three main mechanisms involved: cytokine injury, organ crosstalk, and systemic effects of infection [68].

#### Cytokine injury

Cytokine storm through release of IL-6/JAK2/STAT3/SOCS3 and NF-κB (p65)/IL-18 could work together to induce AKI and increase overall renal-related diagnostic markers [69]. McElvaney OJ et al. showed that IL-1β, IL-6, IL-8, and sTNFR1 were all increased in patients with COVID-19. COVID ICU patients could be clearly differentiated from COVID stable patients, and demonstrated higher levels of IL-1β, IL-6, and sTNFR1 [70]. The contribution of increased vascular permeability and volume depletion, as well as cardiomyopathy—which can lead to type I cardiorenal syndrome—in addition to cytokine activation, is yet to be established. Removing cytokines with extracorporeal therapies, often studied as a promising approach in patients with sepsis and AKI, has been proposed in patients with COVID-19 who develop acute renal failure [7, 71].

#### Organ crosstalk

Connection between alveolar injury and tubular injury has been proven. A retrospective study that included 357 patients with ARDS without kidney disease or AKI at presentation reported that 68% of patients developed AKI. Positive fluid balance, greater disease severity, older patients, and diabetes were independently associated with the development of AKI [72].

#### Systemic effects

Hemodynamic instability associated with rhabdomyolysis, metabolic acidosis, and hyperkalemia can also occur in COVID-19 patients and contribute to AKI.

### Treatment

The indications for renal replacement therapy (RRT) for AKI in critically ill patients are well stablished, regardless of patients' COVID-19 status. However, in an intensive care services overload scenario, providing RRT to an increasing number of patients may exceed the capacity 1 of available machines, supplies, and specialized staff.

In patients without indication of RRT, conservative treatment includes: appropriate dose-loop diuretics (oral or intravenous) for fluid overload and active management of hyperkalemia and metabolic acidosis with potassium binders and sodium bicarbonate. For patients that do not respond to conservative treatment, RRT is indicated.

The use of CRRT remains preferred among critically ill patients with AKI. Even among hemodynamically stable patients who can tolerate intermittent hemodialysis, CRRT, or sustained low-efficiency dialysis (SLED) are preferred, depending on machinery and staff availability and expertise. CRRT or SLED can be managed without 1:1 nursing support, which could potentially help in minimizing the waste of personal protective equipment and limit exposure among nurses on hemodialysis [73].

Regional citrate is the most used anticoagulation strategy during hemofiltration or dialysis. However, some case reports suggest that circuit thrombosis during RRT occurs more frequently in patients with COVID-19 than in other patients, and in these cases, the addition of therapeutic anticoagulation with non-fractioned heparin might be considered [74].

## Metabolic impairment and treatment strategies

Data suggest that diabetes, hypertension, and cardiovascular diseases (CVD) are the most common comorbidities related to COVID-19, although prevalence rates vary among different studies. In a pooled data from 10 Chinese studies, prevalence of hypertension, diabetes, and CVD was 21, 11, and 7%, respectively [75].

Recent data suggest that diabetic patients with COVID-19 are more often associated with most severe forms of the disease, varying between 14 and 32% in different studies, with an odds ratio of 2.34 for ARDS compared with patients without diabetes [10].

A study from Wuhan with 161 patients with COVID-19 demonstrated a delayed viral clearance in patients with diabetes. It has been proposed that, in addition to the usual mechanisms (impaired neutrophil chemotaxis, and phagocytosis) by which diabetes predisposes to infections 1 in general, other specific factors related to SARS-CoV-2 can have roles in the increased risk and severity of the disease in diabetes, as following [76]:

- Increased expression of angiotensin-converting enzyme-2 (ACE2): acute-phase hyperglycemia results in increased ACE2 expression, which can facilitate the entry of viral cells; however, chronic hyperglycemia reduces expression of ACE2, leading to an increased vulnerability to the inflammatory and harmful effects of the virus [77]. In addition, in pancreatic islets, the effect of SARS-CoV on ACE2 blood glucose receptors can lead to hyperglycemia, even in patients without pre-existing diabetes. In patients with SARS-CoV hyperglycemia persisted for up to 3 years after recovery, indicating transient damage to beta cells [78].
- Increased furin: the amount of furin, a membrane-linked protease belonging to the subtilisin/cexinfamily proprotein convertase (PCSK), increases in diabetic individuals. Its role in entry of viruses into the cell, acting as a facilitator for viral replication, has been demonstrated [79].
- Impaired T-cell function: lymphocytopenia was observed in patients with COVID-19 and was correlated with a worse prognosis [80].
- Increased interleukin-6 (IL-6): IL-6 levels are higher in patients with diabetes. Moreover, it is one of the most relevant cytokines activated in cytokine storm in COVID-19 patients. Thus, it may play a more deleterious role in SARS-CoV-2 infection.

Another potential pathway that may explain the correlation between COVID-19 and diabetes involves the enzyme dipeptidyl peptidase-4 (DPP-4), one of the main targets of pharmacological treatment in patients with type 2 diabetes. DPP-4 works as a functional receptor for MERS-CoV in vitro. Despite direct participation of DPP-4 in glucose and insulin metabolism in type 2 diabetes, it has been shown to also increase inflammation. However, a possible role in SARS-CoV-2 infection and whether treatment of diabetes with DPP-4 inhibitors could alter the course of COVID-19 infection are not known yet [81].

### Glycemic control

Monitoring blood glucose levels is an important factor in acute stage and follow-up, especially in those receiving corticosteroid therapy. To date, limited data are available on the association of blood 1 glucose levels and COVID-19; however, data from SARS and H1N1 infections have shown that poor glycemic control increases the risk of complications and death [82].

Thereby, the recommendations for critically ill patients with COVID-19 are:

- Monitor blood glucose in infected patients;
- Glycemic control in patients already known to be diabetic: collection of plasma glucose, electrolytes, and pH;
- Liberal indication for early use of intravenous insulin in severe cases (ARDS and shock), avoiding subcutaneous use;
- Therapeutic objectives:
  - Blood glucose between 72 and 144 mg/dL or 4–16 mmol/L;
  - In elderly patients (> 70 years) or fragile: blood glucose minimum 90 mg/dL or 5 mmol/L [83].

## Specific treatment

Several drugs have been studied for the treatment of the SARS-CoV-2. Most studied antivirals in this scenario were the combination of lopinavir–ritonavir and remdesivir [84, 85]. Currently, the antiviral therapy that appears most promising is remdesivir. This is a prodrug of a nucleotide analogue that is intracellularly metabolized to an analogue of adenosine triphosphate that inhibits viral RNA polymerases. In previous studies, remdesivir was shown to have in vitro activity against Ebola and several coronaviruses, showing a prophylactic and therapeutic efficacy in nonclinical models [86, 87].

Remdesivir has been used recently on a compassionate basis, due to a lack of proven efficacy drugs. A multicenter RCT including 1063 patients receiving remdesivir or placebo showed that remdesivir use led to a significantly shorter duration of hospital stay (11 vs. 15 days), and lower mortality (8% vs. 11.6%) [88]. The FDA has approved this drug for urgent use in COVID-19, it is prescribed intravenously (200 mg IV day 1, and 100 mg IV from day 2 to day 10). A paper published by the same group of authors showed that 5 days of therapy were as effective as 10 days [89].

The antivirals lopinavir/ritonavir, ribavirin, atazanavir, and favipiravir are being tested in the context of COVID-19. Previous in vitro studies suggested that lopinavir presents inhibitory activity against SARS-CoV and MERS-CoV [90–92]. Cao B et al. demonstrated in an RCT with 199 hospitalized patients with respiratory failure that lopinavir/ritonavir did not result in any clinical benefit beyond standard care [84]. A recent multicenter trial tested the combination of lopinavir/ritonavir with interferon beta-1b and ribavirin, and confirmed that in comparison with standard care, the combination of drugs resulted in shorter duration of viral shedding and hospital stay and in clinical improvement [93].

As adjuvant therapies in the treatment of COVID-19, chloroquine and hydroxychloroquine have been evaluated in experimental and clinical studies. These drugs have the ability to increase the endosomal pH of cells and reduce replication of SARS-CoV-2 in vitro [94, 95]. However, initial clinical studies have not revealed any clinical benefit for using these drugs either alone or in combination with azithromycin [96, 97]. Cavalcanti et al. in a randomized clinical trial with 667 hospitalized patients with suspected or confirmed COVID-19 who presented with mild-to-moderate manifestation, the use of hydroxychloroquine, alone or with azithromycin, did not improve clinical status at 15 days as compared with standard care [98]. Similarly, the use of hydroxychloroquine has not been beneficial in preventing the development of COVID-19 in patients after high-risk exposure [99]. The routine use of these drugs is not recommended.

In addition, Mercuro et al., in a cohort of 90 patients, showed that hydroxychloroquine in COVID-19 patients was associated with a high risk of QTc prolongation, and concurrent treatment with azithromycin resulted in greater changes in QTc. We recommend that physicians carefully evaluate the benefits and potential risks of this drug [100].

The use of corticosteroids reduces mortality in COVID-19 patients needing respiratory support [101]. The trial showed that dexamethasone 6 mg once a day for 10 days results in lower 28-day mortality among those who were receiving either invasive mechanical ventilation or oxygen. Usual regimens of steroids usually prescribed in ARDS which might be considered in COVID-19 include intravenous methylprednisolone 0.5 mg/Kg twice a day for 5 days, dexamethasone 6 mg once a day for 10 days or dexamethasone 20 mg once a day for 5 days followed by dexamethasone 10 mg once a day for 5 days [102–104]. A recent meta-analysis included 678 patients who received steroids during COVID-19 (hydrocortisone, dexamethasone, or methylprednisolone) and showed that the administration of systemic corticosteroids, compared with usual care or placebo, was associated with lower 28-day all-cause mortality [105].

Immunomodulatory drugs such as tocilizumab (antihuman IL-6 receptor antibody), sarilumab (anti-IL6 receptor), anakinra (anti-IL1), reparixin (anti-IL8), interferon-α, and complement inhibitors have been explored as potential therapeutic drugs to improve outcomes in COVID-19 patients [106–110].

Tocilizumab use has been associated with reduced mechanical ventilation and reduced serum IL-6 in these patients [109, 110]. The benefit may be related to the phenotype of intense inflammation, characterized by high levels of IL-6, D-dimer, C-reactive protein, LDH, and ferritin. Its preferential use must be carried out through clinical research protocols [111]; patients admitted early to the ICU and still not intubated are probably who mostly benefit. Two retrospective studies showed efficacy of tocilizumab in COVID-19 [112, 113]. However, 4 randomized studies did not confirm the efficacy of tocilizumab in COVID-19 [114–117].

Convalescent plasma has been used for the treatment of infectious diseases since the early twentieth century with reduced mortality in cases series and case reports during the 1918 influenza, the 2003 SARS, and the 2009 influenza H1N13 pandemics [118]. In COVID-19, several uncontrolled case series of convalescent plasma use were performed, suggesting a survival benefit [119, 120].

Li et al. published the first RCT of convalescent plasma in COVID-19, showing no significant benefit in clinical improvement or mortality. However, this study suggests possible benefit in the subgroup of severely ill patients. Further clinical trials are needed to establish the clinical indications for antibody therapies against COVID-19 [98].

### Antithrombotic treatment

Progressive respiratory failure is the primary cause of death in the COVID-19 pandemic, followed by cardiovascular complications. Pathological studies performed in COVID-19 patients showed severe endothelial injury, associated with the presence of intracellular virus and disrupted cell membranes. Pulmonary vessels had widespread thrombosis with microangiopathy and alveolar microthrombi [121]. A Brazilian study of 10 minimally invasive autopsies revealed the presence of diffuse alveolar damage in the lung, and epithelial viral cytopathic effects in alveolar and small airway epithelia. A variable number of small fibrinous thrombi in small pulmonary arterioles were found in areas of both damaged and preserved lungs. Signs of bacterial pneumonia were observed in 6 of 10 cases [122].

In patients who died from COVID-19-associated or influenza-associated respiratory failure, the histologic pattern in the peripheral lung was diffuse alveolar damage with T-cell infiltration. However, increased thrombogenesis was 9 times more prevalent in patients with COVID-19 than in influenza patients [121]. COVID-19 has been described as a thromboinflammatory disease [15] with thrombogenesis a consequence of severe endothelial injury, exacerbated inflammation, suppressed fibrinolysis, loss of natural anticoagulants, and activation of platelets and coagulation factors. Because of these pathophysiologic findings, initial studies started to look for deep venous thrombosis, arterial thrombosis, and microthrombosis. The available data on thrombotic risk are quite limited and based largely on case series from China [123], the Netherlands [6], and France [124]. Recent studies have described a high incidence of deep venous thrombosis and pulmonary embolism varying from 35 to 78% in COVID-19 patients [5, 125, 126].

Nonetheless, most experts agree that the signal for increased thrombotic risk is sufficient to recommend pharmacologic venous thromboembolism (VTE) prophylaxis in all hospitalized COVID-19 patients as long as there is no contraindication [28, 127]. What remains to be confirmed is the real role of therapeutic anticoagulation in these patients. There is much controversy about this issue, while RCT results are not available [16, 128]. The guidelines specifically mention that the anticoagulation regimens may be modified based on extremes of body weight (50% increase in dose if obese), severe thrombocytopenia, or worsening renal function [129].

**Fig. 4:**
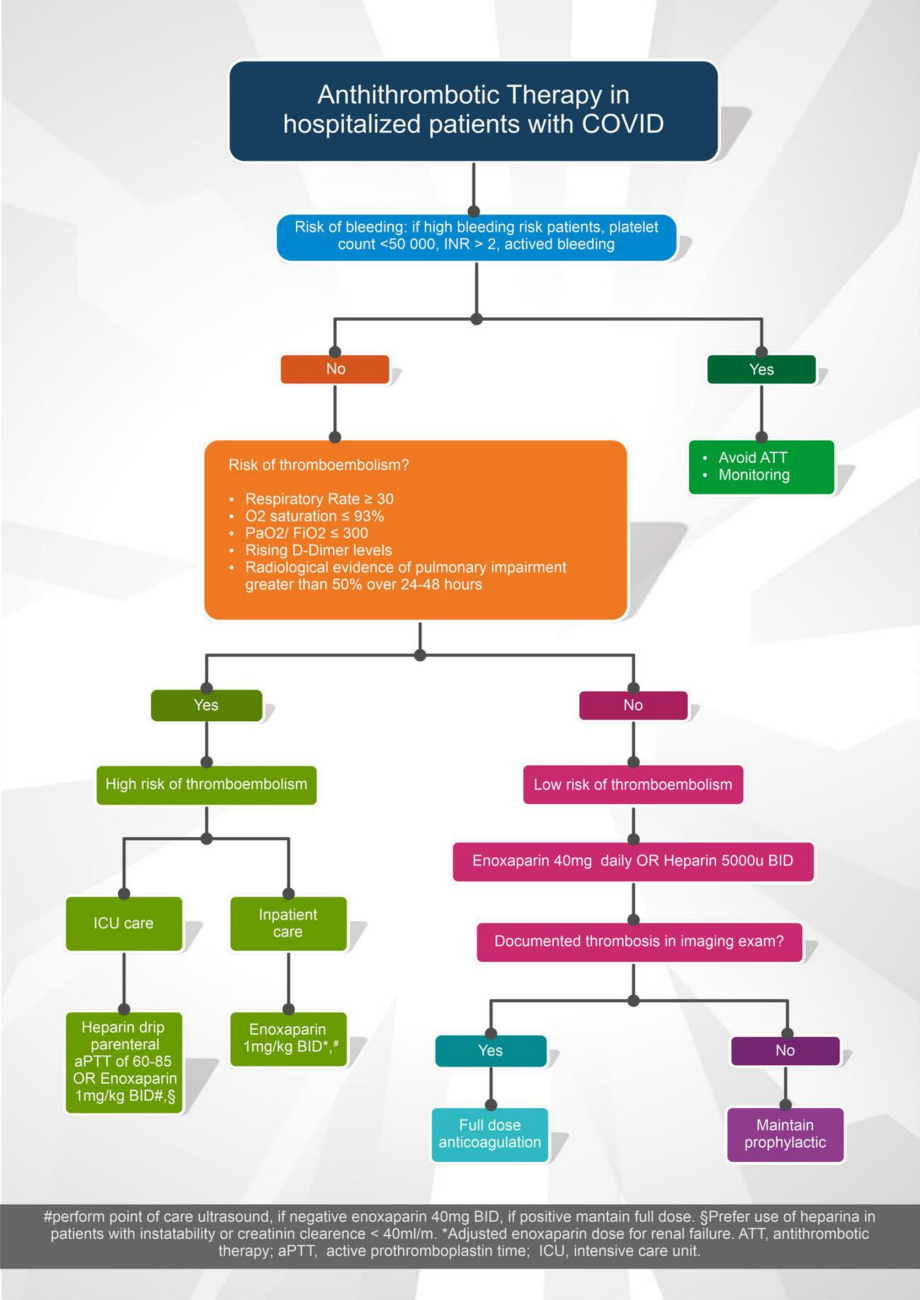
Algorithm proposed for the management of antithrombotic therapy in patients with COVID-19

One of the difficulties in determining the true incidence of thrombosis is that access to diagnostic testing may be limited. In a report from the Netherlands (where routine VTE prophylaxis is given), high rates of VTE were noted among ICU patients [6]. More than one-third of these patients, however, had a pulmonary embolism limited to subsegmental. The therapeutic dose should be considered for patients with severe COVID-19 and signs of 1 sepsis-induced coagulopathy (SIC) and/or high D-dimer (6 × higher the reference values) in association with other biomarkers of severity, in the absence of contraindication for anticoagulation. This can be considered a therapeutic strategy for SARS-CoV-2 infection, based on experts’ opinion and a few retrospective studies [15, 16]. Moreover, this strategy requires the use of strict institutional protocols that enable surveillance and rapid intervention if complications occur. Figure 4 shows a proposed algorithm to assess thrombogenesis in patients with COVID-19, as well as a treatment suggestion. However, data are still insufficient to identify critical aspects relevant to therapeutic plans, such as the best drug choice, its dosage, and administration time schedule, as well as the duration of treatment.

## Ethical dilemma during the pandemics

The COVID-19 epidemic requires an increased number of resources, reinforcement of the ICU teams with new staff members, reorganization of the unit, and protocol changing. All of these might create vulnerability and loss of control for all the professionals. In many times, intensivists should make their choices based on local politics, structural resources, and team abilities [130].

## Conclusions

COVID-19 is one of the most challenging health emergencies we have faced this century. Health professionals are experiencing very difficult times, with limitations of resources and information, which when available needs to be confirmed before integration into clinical practice. To date, there is no proven specific treatment for the management of COVID-19, although studies with promising results have recently emerged. We know already that the lowest rates of mortality are related to better quality of care. Early diagnosis, application of effective therapies and adequate strategies of clinical stratification are needed for better outcomes in COVID-19 patients. We have learned that COVID-19-related respiratory dysfunction has unique characteristics that require individualized management and are aware of the importance of supporting the patients’ hemodynamics because of the high risk of cardiovascular and renal complications. The long duration of this disease poses a challenge for the health system and health professionals. Validated protocols 1 of care are essential when dealing with millions of affected people in different countries and in different levels of care. Until an effective vaccine is widely available, the world will need to adapt to the reality of a pandemic that has come to change all paradigms of modern medicine.

## Data Availability

Not applicable.

## Abbreviations

ACE2: Angiotensin-converting enzyme-2
ACP: Acute Cor Pulmonale
AKI: Acute kidney injury
AKIN: Acute Kidney Injury Net
ARDS: Acute respiratory distress syndrome
BIPAP: Nasal bilevel positive airway pressure
BGA: Blood gas analysis
COPD: Chronic obstructive pulmonary disease
COVID-19: Coronavirus disease 2019
CRRT: Continuous renal replacement therapy
CVD: Cardiovascular diseases.
DPP-4: Dipeptidyl peptidase-4
ECMO: Extracorporeal membrane oxygenation
ICU: Intensive care units
IL: Interleukin.
LDH: Lactate dehydrogenase
LV: Left ventricular
NIV: Noninvasive ventilation
PEEP: Positive end-expiratory pressure
P-SILI: Self-induced lung injury
RCT: Randomized clinical trial
RRT: Renal replacement therapy
RV: Right ventricular
SARS-CoV-2: Severe acute respiratory syndrome coronavirus 2
SLED: Sustained low-efficiency dialysis
VV-ECMO: Venovenous extracorporeal membrane oxygenation
VT: Tidal volumes
VTE: Venous thromboembolism

## References

[1] Malik P, Patel U, Mehta D, Patel N, Kelkar R, Akrmah M (2020). Biomarkers and outcomes of COVID-19 hospitalisations: systematic review and meta-analysis. BMJ Evid Based Med..

[2] Vincent JL, Slutsky AS (2020). Coronavirus: just imagine. Crit Care.

[3] Wiersinga WJ, Rhodes A, Cheng AC, Peacock SJ, Prescott HC (2020). Pathophysiology, transmission, diagnosis, and treatment of Coronavirus Disease 2019 (COVID-19): a review. JAMA.

[4] Costa I, Bittar CS, Rizk SI, Araújo Filho AE, Santos KAQ, Machado TIV (2020). The heart and COVID-19: what cardiologists need to know. Arq Bras Cardiol.

[5] Fraisse M, Logre E, Pajot O, Mentec H, Plantefeve G, Contou D (2020). Thrombotic and hemorrhagic events in critically ill COVID-19 patients: a French monocenter retrospective study. Crit Care.

[6] Klok FA, Kruip M, van der Meer NJM, Arbous MS, Gommers D, Kant KM (2020). Incidence of thrombotic complications in critically ill ICU patients with COVID-19. Thromb Res.

[7] Ronco C, Reis T, Husain-Syed F (2020). Management of acute kidney injury in patients with COVID-19. Lancet Respir Med.

[8] Chen YT, Shao SC, Lai EC, Hung MJ, Chen YC (2020). Mortality rate of acute kidney injury in SARS, MERS, and COVID-19 infection: a systematic review and meta-analysis. Crit Care.

[9] Wu Z, McGoogan JM (2020). Characteristics of and important lessons from the Coronavirus Disease 2019 (COVID-19) outbreak in China: summary of a report of 72314 cases from the Chinese center for disease control and prevention. JAMA.

[10] Wu C, Chen X, Cai Y, Xia J, Zhou X, Xu S (2020). Risk factors associated with acute respiratory distress syndrome and death in patients with Coronavirus Disease 2019 Pneumonia in Wuhan, China. JAMA Intern Med..

[11] Phua J, Weng L, Ling L, Egi M, Lim CM, Divatia JV (2020). Intensive care management of coronavirus disease 2019 (COVID-19): challenges and recommendations. Lancet Respir Med.

[12] Zangrillo A, Beretta L, Silvani P, Colombo S, Scandroglio AM, Dell’Acqua A, Fominskiy E, Landoni G, Monti G, Azzolini ML, Monaco F, Oriani A, Belletti A, Sartorelli M, Pallanch O, Saleh O, Sartini C, Nardelli P, Lombardi G, Morselli F, Scquizzato T, Frontera A, Ruggeri A, Scotti R, Assanelli A, Dagna L, Rovere-Querini P, Castagna A, Scarpellini P, Di Napoli D, Ambrosio A, Ciceri F, Tresoldi M (2020). Fast reshaping of intensive care unit facilities in a large metropolitan hospital in Milan, Italy: facing the COVID-19 pandemic emergency. Crit Care Resusc..

[13] Camporota L, Vasques F, Sanderson B, Barrett NA, Gattinoni L (2020). Identification of pathophysiological patterns for triage and respiratory support in COVID-19. Lancet Respir Med.

[14] Diehl JL, Peron N, Chocron R, Debuc B, Guerot E, Hauw-Berlemont C (2020). Respiratory mechanics and gas exchanges in the early course of COVID-19 ARDS: a hypothesis-generating study. Ann Intensive Care.

[15] Ciceri F, Beretta L, Scandroglio AM, Colombo S, Landoni G, Ruggeri A, Peccatori J, D'Angelo A, De Cobelli F, Rovere-Querini P, Tresoldi M, Dagna L, Zangrillo A (2020). Microvascular COVID-19 lung vessels obstructive thromboinflammatory syndrome (MicroCLOTS): an atypical acute respiratory distress syndrome working hypothesis. Crit Care Resusc..

[16] Nascimento JHP, Gomes BFO, Carmo Júnior PR (2020). COVID-19 and hypercoagulable state: a new therapeutic perspective. Arq Bras Cardiol..

[17] Wichmann D, Sperhake JP, Lutgehetmann M, Steurer S, Edler C, Heinemann A (2020). Autopsy findings and venous thromboembolism in patients with COVID-19. Ann Intern Med..

[18] Shi S, Qin M, Shen B, Cai Y, Liu T, Yang F (2020). Association of cardiac injury with mortality in hospitalized patients with COVID-19 in Wuhan, China. JAMA Cardiol..

[19] Garg S, Kim L, Whitaker M, O'Halloran A, Cummings C, Holstein R (2020). Hospitalization rates and characteristics of patients hospitalized with laboratory-confirmed Coronavirus Disease 2019—COVID-NET, 14 States, March 1–30, 2020. MMWR Morb Mortal Wkly Rep.

[20] Rice TW, Wheeler AP, Bernard GR, Hayden DL, Schoenfeld DA, Ware LB (2007). Comparison of the SpO2/FIO2 ratio and the PaO2/FIO2 ratio in patients with acute lung injury or ARDS. Chest.

[21] Gattinoni L, Chiumello D, Caironi P, Busana M, Romitti F, Brazzi L, Camporota L (2020). COVID-19 pneumonia: different respiratory treatments for different phenotypes?. Intensive Care Med..

[22] Alhazzani W, Møller MH, Arabi YM, Loeb M, Gong MN, Fan E, Oczkowski S, Levy MM, Derde L, Dzierba A, Du B, Aboodi M, Wunsch H, Cecconi M, Koh Y, Chertow DS, Maitland K, Alshamsi F, Belley-Cote E, Greco M, Laundy M, Morgan JS, Kesecioglu J, McGeer A, Mermel L, Mammen MJ, Alexander PE, Arrington A, Centofanti JE, Citerio G, Baw B, Memish ZA, Hammond N, Hayden FG, Evans L, Rhodes A (2020). Surviving Sepsis Campaign: guidelines on the management of critically ill adults with Coronavirus Disease 2019 (COVID-19). Intensive Care Med..

[23] Iliescu CA, Grines CL, Herrmann J, Yang EH, Cilingiroglu M, Charitakis K (2016). SCAI Expert consensus statement: Evaluation, management, and special considerations of cardio-oncology patients in the cardiac catheterization laboratory (endorsed by the cardiological society of india, and sociedad Latino Americana de Cardiologia intervencionista). Catheter Cardiovasc Interv.

[24] Padrão EMH, Valente FS, Besen B, Rahhal H, Mesquita PS, de Alencar JCG (2020). Awake prone positioning in COVID-19 hypoxemic respiratory failure: exploratory findings in a single-center retrospective cohort study. Acad Emerg Med.

[25] Elharrar X, Trigui Y, Dols AM, Touchon F, Martinez S, Prud'homme E (2020). Use of prone positioning in nonintubated patients with COVID-19 and hypoxemic acute respiratory failure. JAMA.

[26] Coppo A, Bellani G, Winterton D, Di Pierro M, Soria A, Faverio P (2020). Feasibility and physiological effects of prone positioning in non-intubated patients with acute respiratory failure due to COVID-19 (PRON-COVID): a prospective cohort study. Lancet Respir Med.

[27] Force ADT, Ranieri VM, Rubenfeld GD, Thompson BT, Ferguson ND, Caldwell E (2012). Acute respiratory distress syndrome: the Berlin Definition. JAMA.

[28] Gattinoni L, Marini JJ, Pesenti A, Quintel M, Mancebo J, Brochard L (2016). The, “baby lung” became an adult. Intensive Care Med.

[29] Navas-Blanco JR, Dudaryk R (2020). Management of respiratory distress syndrome due to COVID-19 infection. BMC Anesthesiol.

[30] Shelhamer M, Wesson PD, Solari IL, Jensen DL, Steele WA, Dimitrov VG, Kelly JD, Aziz S, Gutierrez VP, Vittinghoff E, Chung KK, Menon VP, Ambris HA, Baxi SM (2021). Prone Positioning in Moderate to Severe Acute Respiratory Distress Syndrome due to COVID-19: A Cohort Study and Analysis of Physiology. J Intensive Care Med..

[31] Marini JJ, Gattinoni L (2020). Management of COVID-19 respiratory distress. JAMA.

[32] Sorbello M, El-Boghdadly K, Di Giacinto I, Cataldo R, Esposito C, Falcetta S (2020). The Italian coronavirus disease 2019 outbreak: recommendations from clinical practice. Anaesthesia.

[33] Grasselli G, Zangrillo A, Zanella A, Antonelli M, Cabrini L, Castelli A (2020). Baseline characteristics and outcomes of 1591 patients infected with SARS-CoV-2 admitted to ICUs of the Lombardy Region, Italy. JAMA.

[34] Zangrillo A, Beretta L, Scandroglio AM, Monti G, Fominskiy E, Colombo S, Morselli F, Belletti A, Silvani P, Crivellari M, Monaco F, Azzolini ML, Reineke R, Nardelli P, Sartorelli M, Votta CD, Ruggeri A, Ciceri F, De Cobelli F, Tresoldi M, Dagna L, Rovere-Querini P, Serpa Neto A, Bellomo R, Landoni G (2020). COVID-BioB Study Group. Characteristics, treatment, outcomes and cause of death of invasively ventilated patients with COVID-19 ARDS in Milan, Italy. Crit Care Resusc.

[35] Driggin E, Madhavan MV, Bikdeli B, Chuich T, Laracy J, Bondi-Zoccai G (2020). Cardiovascular considerations for patients, health care workers, and health systems during the Coronavirus Disease 2019 (COVID-19) pandemic. J Am Coll Cardiol..

[36] Tissieres P, Teboul JL (2020). SARS-CoV-2 post-infective myocarditis: the tip of COVID-19 immune complications?. Ann Intensive Care.

[37] Szekely Y, Lichter Y, Taieb P, Banai A, Hochstadt A, Merdler I (2020). The spectrum of cardiac manifestations in Coronavirus Disease 2019 (COVID-19)—a Systematic Echocardiographic Study. Circulation.

[38] Yang X, Yu Y, Xu J, Shu H, Xia J, Liu H (2020). Clinical course and outcomes of critically ill patients with SARS-CoV-2 pneumonia in Wuhan, China: a single-centered, retrospective, observational study. Lancet Respir Med.

[39] Wang D, Hu B, Hu C, Zhu F, Liu X, Zhang J (2020). Clinical characteristics of 138 hospitalized patients with 2019 Novel Coronavirus-Infected Pneumonia in Wuhan, China. JAMA.

[40] Goyal P, Choi JJ, Pinheiro LC, Schenck EJ, Chen R, Jabri A (2020). Clinical characteristics of Covid-19 in New York City. N Engl J Med..

[41] McGonagle DODJ, Sharif K, Emery P, Bridgewood C (2020). Immune mechanisms of pulmonary intravascular coagulopathy in COVID-19 pneumonia. Lancet Rheumatol.

[42] Vieillard-Baron A, Matthay M, Teboul JL, Bein T, Schultz M, Magder S (2016). Experts' opinion on management of hemodynamics in ARDS patients: focus on the effects of mechanical ventilation. Intensive Care Med.

[43] Poissy J, Goutay J, Caplan M, Parmentier E, Duburcq T, Lassalle F (2020). Pulmonary embolism in COVID-19 patients: awareness of an increased prevalence. Circulation.

[44] Creel-Bulos C, Hockstein M, Amin N, Melhem S, Truong A, Sharifpour M (2020). Acute cor pulmonale in critically ill patients with Covid-19. N Engl J Med.

[45] Vieillard-Baron A, Girou E, Valente E, Brun-Buisson C, Jardin F, Lemaire F (2000). Predictors of mortality in acute respiratory distress syndrome. Focus on the role of right heart catheterization. Am J Respir Crit Care Med..

[46] National Heart L, Blood Institute Acute Respiratory Distress Syndrome Clinical Trials N, Wiedemann HP, Wheeler AP, Bernard GR, Thompson BT, et al. Comparison of two fluid-management strategies in acute lung injury. N Engl J Med. 2006;354(24):2564–75. 10.1056/NEJMoa062200 16714767

[47] Malbrain M, Van Regenmortel N, Saugel B, De Tavernier B, Van Gaal PJ, Joannes-Boyau O (2018). Principles of fluid management and stewardship in septic shock: it is time to consider the four D's and the four phases of fluid therapy. Ann Intensive Care.

[48] Myatra SN, Prabu NR, Divatia JV, Monnet X, Kulkarni AP, Teboul JL (2017). The changes in pulse pressure variation or stroke volume variation after a “Tidal Volume Challenge” reliably predict fluid responsiveness during low tidal volume ventilation. Crit Care Med.

[49] Kang Y, Chen T, Mui D, Ferrari V, Jagasia D, Scherrer-Crosbie M (2020). Cardiovascular manifestations and treatment considerations in covid-19. Heart.

[50] Zeng JH, Liu YX, Yuan J, Wang FX, Wu WB, Li JX, Wang LF, Gao H, Wang Y, Dong CF, Li YJ, Xie XJ, Feng C, Liu L (2020). First case of COVID-19 complicated with fulminant myocarditis: a case report and insights. Infection..

[51] Puntmann VO, Carerj ML, Wieters I, Fahim M, Arendt C, Hoffmann J (2020). Outcomes of cardiovascular magnetic resonance imaging in patients recently recovered from Coronavirus Disease 2019 (COVID-19). JAMA Cardiol..

[52] Douglas IS, Alapat PM, Corl KA, Exline MC, Forni LG, Holder AL (2020). Fluid response evaluation in sepsis hypotension and shock: a randomized clinical trial. Chest.

[53] Michard F, Boussat S, Chemla D, Anguel N, Mercat A, Lecarpentier Y (2000). Relation between respiratory changes in arterial pulse pressure and fluid responsiveness in septic patients with acute circulatory failure. Am J Respir Crit Care Med.

[54] Yang X, Du B (2014). Does pulse pressure variation predict fluid responsiveness in critically ill patients? A systematic review and meta-analysis. Crit Care.

[55] Poston JT, Patel BK, Davis AM (2020). Management of critically ill adults with COVID-19. JAMA.

[56] McIntyre WF, Um KJ, Alhazzani W, Lengyel AP, Hajjar L, Gordon AC (2018). Association of vasopressin plus catecholamine vasopressors vs catecholamines alone with atrial fibrillation in patients with distributive shock: a systematic review and meta-analysis. JAMA.

[57] Zangrillo A, Landoni G, Beretta L, Morselli F, Serpa Neto A, Bellomo R (2020). Angiotensin II infusion in COVID-19-associated vasodilatory shock: a case series. Crit Care.

[58] Vieillard-Baron A, Charron C, Caille V, Belliard G, Page B, Jardin F (2007). Prone positioning unloads the right ventricle in severe ARDS. Chest.

[59] Dzierba AL, Abel EE, Buckley MS, Lat I (2014). A review of inhaled nitric oxide and aerosolized epoprostenol in acute lung injury or acute respiratory distress syndrome. Pharmacotherapy.

[60] Ronco C, Navalesi P, Vincent JL (2020). Coronavirus epidemic: preparing for extracorporeal organ support in intensive care. Lancet Respir Med.

[61] Bemtgen X, Zotzmann V, Benk C, Rilinger J, Steiner K, Asmussen A (2020). Thrombotic circuit complications during venovenous extracorporeal membrane oxygenation in COVID-19. J Thromb Thrombolysis..

[62] Joseph A, Zafrani L, Mabrouki A, Azoulay E, Darmon M (2020). Acute kidney injury in patients with SARS-CoV-2 infection. Ann Intensive Care.

[63] Chu KH, Tsang WK, Tang CS, Lam MF, Lai FM, To KF (2005). Acute renal impairment in coronavirus-associated severe acute respiratory syndrome. Kidney Int.

[64] Fominskiy EV, Scandroglio AM, Monti G, Calabrò MG, Landoni G, Dell'Acqua A, Beretta L, Moizo E, Ravizza A, Monaco F, Campochiaro C, Pieri M, Azzolini ML, Borghi G, Crivellari M, Conte C, Mattioli C, Silvani P, Mucci M, Turi S, Tentori S, Baiardo Redaelli M, Sartorelli M, Angelillo P, Belletti A, Nardelli P, Nisi FG, Valsecchi G, Barberio C, Ciceri F, Serpa Neto A, Dagna L, Bellomo R, Zangrillo A (2021). COVID-BioB Study Group. Prevalence, Characteristics, Risk Factors, and Outcomes of Invasively Ventilated COVID-19 Patients with Acute Kidney Injury and Renal Replacement Therapy. Blood Purif..

[65] Cheng Y, Luo R, Wang K, Zhang M, Wang Z, Dong L (2020). Kidney disease is associated with in-hospital death of patients with COVID-19. Kidney Int.

[66] Su H, Yang M, Wan C, Yi LX, Tang F, Zhu HY (2020). Renal histopathological analysis of 26 postmortem findings of patients with COVID-19 in China. Kidney Int..

[67] Yin WZP (2020). Infectious pathways of SARS-CoV-2 in renal tissue. J Nephopathol.

[68] Singh AK, Gupta R, Ghosh A, Misra A (2020). Diabetes in COVID-19: Prevalence, pathophysiology, prognosis and practical considerations. Diabetes Metab Syndr.

[69] Saad Alharbi K, Al-Abbasi FA, Prasad Agrawal G, Sharma A, Kowti R, Kazmi I (2020). Impact of COVID-19 on Nephrology Patients: A Mechanistic Outlook for Pathogenesis of Acute Kidney Injury. Altern Ther Health Med..

[70] McElvaney OJ, McEvoy NL, McElvaney OF, Carroll TP, Murphy MP, Dunlea DM, Ní Choileáin O, Clarke J, O'Connor E, Hogan G, Ryan D, Sulaiman I, Gunaratnam C, Branagan P, O'Brien ME, Morgan RK, Costello RW, Hurley K, Walsh S, de Barra E, McNally C, McConkey S, Boland F, Galvin S, Kiernan F, O'Rourke J, Dwyer R, Power M, Geoghegan P, Larkin C, O'Leary RA, Freeman J, Gaffney A, Marsh B, Curley GF, McElvaney NG (2020). Characterization of the Inflammatory Response to Severe COVID-19 Illness. Am J Respir Crit Care Med..

[71] Ronco C, Reis T, De Rosa S (2020). Coronavirus epidemic and extracorporeal therapies in intensive Care: si vis pacem para bellum. Blood Purif.

[72] Panitchote A, Mehkri O, Hastings A, Hanane T, Demirjian S, Torbic H (2019). Factors associated with acute kidney injury in acute respiratory distress syndrome. Ann Intensive Care.

[73] Gallieni M, Sabiu G, Scorza D (2020). Delivering safe and effective hemodialysis in patients with suspected or confirmed COVID-19 infection: a single-center perspective from Italy. Kidney.

[74] Improvement NEaN**.** Specialty guides for patient management during the coronavirus pandemic Clinical guide for renal replacement therapy options in critical care during the coronavirus pandemic https://www.england.nhs.uk/. 2020.

[75] Singh AK, Gupta R, Misra A (2020). Comorbidities in COVID-19: outcomes in hypertensive cohort and controversies with renin angiotensin system blockers. Diabetes Metab Syndr.

[76] Trump S, Lukassen S, Anker MS, Chua RL, Liebig J, Thürmann L, Corman VM, Binder M, Loske J, Klasa C, Krieger T, Hennig BP, Messingschlager M, Pott F, Kazmierski J, Twardziok S, Albrecht JP, Eils J, Hadzibegovic S, Lena A, Heidecker B, Bürgel T, Steinfeldt J, Goffinet C, Kurth F, Witzenrath M, Völker MT, Müller SD, Liebert UG, Ishaque N, Kaderali L, Sander LE, Drosten C, Laudi S, Eils R, Conrad C, Landmesser U, Lehmann I (2020). Hypertension delays viral clearance and exacerbates airway hyperinflammation in patients with COVID-19. Nat Biotechnol..

[77] Bindom SM, Lazartigues E (2009). The sweeter side of ACE2: physiological evidence for a role in diabetes. Mol Cell Endocrinol.

[78] Yang JK, Lin SS, Ji XJ, Guo LM (2010). Binding of SARS coronavirus to its receptor damages islets and causes acute diabetes. Acta Diabetol.

[79] Fernandez C, Rysä J, Almgren P, Nilsson J, Engström G, Orho-Melander M (2018). Plasma levels of the proprotein convertase furin and incidence of diabetes and mortality. J Intern Med.

[80] Guan WJ, Ni ZY, Hu Y, Liang WH, Ou CQ, He JX (2020). Clinical characteristics of Coronavirus Disease 2019 in China. N Engl J Med.

[81] Iacobellis G (2020). COVID-19 and diabetes: can DPP4 inhibition play a role?. Diabetes Res Clin Pract.

[82] Yang JK, Feng Y, Yuan MY, Yuan SY, Fu HJ, Wu BY (2006). Plasma glucose levels and diabetes are independent predictors for mortality and morbidity in patients with SARS. Diabet Med.

[83] Bornstein SR, Rubino F, Khunti K, Mingrone G, Hopkins D, Birkenfeld AL (2020). Practical recommendations for the management of diabetes in patients with COVID-19. Lancet Diabetes Endocrinol..

[84] Cao B, Wang Y, Wen D, Liu W, Wang J, Fan G (2020). A Trial of lopinavir-ritonavir in adults hospitalized with severe Covid-19. N Engl J Med.

[85] Grein J, Ohmagari N, Shin D, Diaz G, Asperges E, Castagna A, Feldt T, Green G, Green ML, Lescure FX, Nicastri E, Oda R, Yo K, Quiros-Roldan E, Studemeister A, Redinski J, Ahmed S, Bernett J, Chelliah D, Chen D, Chihara S, Cohen SH, Cunningham J, D'Arminio Monforte A, Ismail S, Kato H, Lapadula G, L'Her E, Maeno T, Majumder S, Massari M, Mora-Rillo M, Mutoh Y, Nguyen D, Verweij E, Zoufaly A, Osinusi AO, DeZure A, Zhao Y, Zhong L, Chokkalingam A, Elboudwarej E, Telep L, Timbs L, Henne I, Sellers S, Cao H, Tan SK, Winterbourne L, Desai P, Mera R, Gaggar A, Myers RP, Brainard DM, Childs R, Flanigan T (2020). Compassionate Use of Remdesivir for Patients with Severe Covid-19. N Engl J Med..

[86] de Wit E, Feldmann F, Cronin J, Jordan R, Okumura A, Thomas T (2020). Prophylactic and therapeutic remdesivir (GS-5734) treatment in the rhesus macaque model of MERS-CoV infection. Proc Natl Acad Sci USA.

[87] Sheahan TP, Sims AC, Leist SR, Schäfer A, Won J, Brown AJ (2020). Comparative therapeutic efficacy of remdesivir and combination lopinavir, ritonavir, and interferon beta against MERS-CoV. Nat Commun.

[88] Beigel JH, Tomashek KM, Dodd LE, Mehta AK, Zingman BS, Kalil AC, Hohmann E, Chu HY, Luetkemeyer A, Kline S, Lopez de Castilla D, Finberg RW, Dierberg K, Tapson V, Hsieh L, Patterson TF, Paredes R, Sweeney DA, Short WR, Touloumi G, Lye DC, Ohmagari N, Oh MD, Ruiz-Palacios GM, Benfield T, Fätkenheuer G, Kortepeter MG, Atmar RL, Creech CB, Lundgren J, Babiker AG, Pett S, Neaton JD, Burgess TH, Bonnett T, Green M, Makowski M, Osinusi A, Nayak S, Lane HC (2020). ACTT-1 Study Group Members. Remdesivir for the Treatment of Covid-19 - Final Report. N Engl J Med..

[89] Goldman JD, Lye DCB, Hui DS, Marks KM, Bruno R, Montejano R (2020). Remdesivir for 5 or 10 days in patients with severe Covid-19. N Engl J Med..

[90] Chu CM, Cheng VC, Hung IF, Wong MM, Chan KH, Chan KS (2004). Role of lopinavir/ritonavir in the treatment of SARS: initial virological and clinical findings. Thorax.

[91] Chen F, Chan KH, Jiang Y, Kao RY, Lu HT, Fan KW (2004). In vitro susceptibility of 10 clinical isolates of SARS coronavirus to selected antiviral compounds. J Clin Virol.

[92] de Wilde AH, Jochmans D, Posthuma CC, Zevenhoven-Dobbe JC, van Nieuwkoop S, Bestebroer TM (2014). Screening of an FDA-approved compound library identifies four small-molecule inhibitors of Middle East respiratory syndrome coronavirus replication in cell culture. Antimicrob Agents Chemother.

[93] Hung IF, Lung KC, Tso EY, Liu R, Chung TW, Chu MY (2020). Triple combination of interferon beta-1b, lopinavir-ritonavir, and ribavirin in the treatment of patients admitted to hospital with COVID-19: an open-label, randomised, phase 2 trial. Lancet.

[94] Gao J, Tian Z, Yang X (2020). Breakthrough: Chloroquine phosphate has shown apparent efficacy in treatment of COVID-19 associated pneumonia in clinical studies. Biosci Trends.

[95] Yao X, Ye F, Zhang M, Cui C, Huang B, Niu P (2020). In vitro antiviral activity and projection of optimized dosing design of hydroxychloroquine for the treatment of severe acute respiratory syndrome Coronavirus 2 (SARS-CoV-2). Clin Infect Dis..

[96] Geleris J, Sun Y, Platt J, Zucker J, Baldwin M, Hripcsak G (2020). Observational study of hydroxychloroquine in hospitalized patients with Covid-19. N Engl J Med..

[97] Rosenberg ES, Dufort EM, Udo T, Wilberschied LA, Kumar J, Tesoriero J (2020). Association of treatment with hydroxychloroquine or azithromycin with in-hospital mortality in patients with COVID-19 in New York State. JAMA.

[98] Cavalcanti AB, Zampieri FG, Rosa RG, Azevedo LCP, Veiga VC, Avezum A, Damiani LP, Marcadenti A, Kawano-Dourado L, Lisboa T, Junqueira DLM, de Barros E Silva PGM, Tramujas L, Abreu-Silva EO, Laranjeira LN, Soares AT, Echenique LS, Pereira AJ, Freitas FGR, Gebara OCE, Dantas VCS, Furtado RHM, Milan EP, Golin NA, Cardoso FF, Maia IS, Hoffmann Filho CR, Kormann APM, Amazonas RB, Bocchi de Oliveira MF, Serpa-Neto A, Falavigna M, Lopes RD, Machado FR, Berwanger O; Coalition Covid-19 Brazil I Investigators. Hydroxychloroquine with or without Azithromycin in Mild-to-Moderate Covid-19. N Engl J Med. 2020;383(21):2041–52. 10.1056/NEJMoa2019014**[Erratum in: N Engl J Med. 2020 Nov 19;383(21):e119]**.

[99] Boulware DR, Pullen MF, Bangdiwala AS, Pastick KA, Lofgren SM, Okafor EC (2020). A randomized trial of hydroxychloroquine as postexposure prophylaxis for Covid-19. N Engl J Med.

[100] Mercuro NJ, Yen CF, Shim DJ, Maher TR, McCoy CM, Zimetbaum PJ (2020). Risk of QT interval prolongation associated with use of hydroxychloroquine with or without concomitant azithromycin among hospitalized patients testing positive for Coronavirus Disease 2019 (COVID-19). JAMA Cardiol..

[101] RECOVERY Collaborative Group, Horby P, Lim WS, Emberson JR, Mafham M, Bell JL, Linsell L, Staplin N, Brightling C, Ustianowski A, Elmahi E, Prudon B, Green C, Felton T, Chadwick D, Rege K, Fegan C, Chappell LC, Faust SN, Jaki T, Jeffery K, Montgomery A, Rowan K, Juszczak E, Baillie JK, Haynes R, Landray MJ. Dexamethasone in Hospitalized Patients with Covid-19 - Preliminary Report. N Engl J Med. 2020;NEJMoa2021436. 10.1056/NEJMoa2021436.

[102] Meduri GU, Chrousos GP (2020). General adaptation in critical illness: glucocorticoid receptor-alpha master regulator of homeostatic corrections. Front Endocrinol (Lausanne).

[103] Villar J, Ferrando C, Martínez D, Ambrós A, Muñoz T, Soler JA (2020). Dexamethasone treatment for the acute respiratory distress syndrome: a multicentre, randomised controlled trial. Lancet Respir Med.

[104] Tomazini BM, Maia IS, Cavalcanti AB, Berwanger O, Rosa RG, Veiga VC (2020). Effect of dexamethasone on days alive and ventilator-free in patients with moderate or severe acute respiratory distress syndrome and COVID-19: the CoDEX Randomized Clinical Trial. JAMA.

[105] Group WHOREAfC-TW, Sterne JAC, Murthy S, Diaz JV, Slutsky AS, Villar J, et al. Association Between Administration of Systemic Corticosteroids and Mortality Among Critically Ill Patients With COVID-19: A Meta-analysis. JAMA. 2020;324(13):1330–41. 10.1001/jama.2020.17023 PMC7489434 32876694

[106] Cavalli GDLG, Campochiaro C, Della Torre E, Ripa M, Canetti D, Oltolini C, Castiglioni B (2020). Interleukin 1 blockade with high dose intravenous anakinra in patients with COVID-19, acute respiratory distress syndrome, and hyper-inflammation: a retrospective cohort study. Lancet Rheumatol..

[107] Risitano AM, Mastellos DC, Huber-Lang M, Yancopoulou D, Garlanda C, Ciceri F (2020). Complement as a target in COVID-19?. Nat Rev Immunol.

[108] Mastaglio S, Ruggeri A, Risitano AM, Angelillo P, Yancopoulou D, Mastellos DC (2020). The first case of COVID-19 treated with the complement C3 inhibitor AMY-101. Clin Immunol.

[109] Xu X, Han M, Li T, Sun W, Wang D, Fu B (2020). Effective treatment of severe COVID-19 patients with tocilizumab. Proc Natl Acad Sci USA.

[110] Luo P, Liu Y, Qiu L, Liu X, Liu D, Li J (2020). Tocilizumab treatment in COVID-19: a single center experience. J Med Virol..

[111] Bhimraj A MR, Shumaker AH, Lavergne V, Baden L, Cheng VCC, et al. Infectious Diseases Society of America Guidelines on the treatment and management of patients with COVID-19 infection. www.idsociety.org/COVID19guidelines. 2020. 10.1093/cid/ciaa478 PMC7197612 32338708

[112] Guaraldi G, Meschiari M, Cozzi-Lepri A, Milic J, Tonelli R, Menozzi M (2020). Tocilizumab in patients with severe COVID-19: a retrospective cohort study. Lancet Rheumatol.

[113] Biran N, Ip A, Ahn J, Go RC, Wang S, Mathura S (2020). Tocilizumab among patients with COVID-19 in the intensive care unit: a multicentre observational study. Lancet Rheumatol.

[114] Stone JH, Frigault MJ, Serling-Boyd NJ, Fernandes AD, Harvey L, Foulkes AS (2020). Efficacy of tocilizumab in patients hospitalized with Covid-19. N Engl J Med..

[115] Hermine O, Mariette X, Tharaux PL, Resche-Rigon M, Porcher R, Ravaud P (2021). CORIMUNO-19 Collaborative Group. Effect of Tocilizumab vs Usual Care in Adults Hospitalized With COVID-19 and Moderate or Severe Pneumonia: A Randomized Clinical Trial. JAMA. Intern Med..

[116] Salvarani C, Dolci G, Massari M, Merlo DF, Cavuto S, Savoldi L, Bruzzi P, Boni F, Braglia L, Turrà C, Ballerini PF, Sciascia R, Zammarchi L, Para O, Scotton PG, Inojosa WO, Ravagnani V, Salerno ND, Sainaghi PP, Brignone A, Codeluppi M, Teopompi E, Milesi M, Bertomoro P, Claudio N, Salio M, Falcone M, Cenderello G, Donghi L, Del Bono V, Colombelli PL, Angheben A, Passaro A, Secondo G, Pascale R, Piazza I, Facciolongo N, Costantini M (2021). RCT-TCZ-COVID-19 Study Group. Effect of Tocilizumab vs Standard Care on Clinical Worsening in Patients Hospitalized With COVID-19 Pneumonia: a Randomized Clinical Trial. JAMA. Intern Med..

[117] Gupta S, Hayek SS, Wang W, Chan L, Mathews KS, Melamed ML (2020). Factors associated with death in critically ill patients with Coronavirus Disease 2019 in the US. JAMA Intern Med..

[118] Casadevall A, Joyner MJ, Pirofski LA (2020). A randomized trial of convalescent plasma for COVID-19-potentially hopeful signals. JAMA.

[119] Duan K, Liu B, Li C, Zhang H, Yu T, Qu J (2020). Effectiveness of convalescent plasma therapy in severe COVID-19 patients. Proc Natl Acad Sci USA.

[120] Shen C, Wang Z, Zhao F, Yang Y, Li J, Yuan J (2020). Treatment of 5 critically ill patients with COVID-19 with convalescent plasma. JAMA.

[121] Ackermann M, Verleden SE, Kuehnel M, Haverich A, Welte T, Laenger F, Vanstapel A, Werlein C, Stark H, Tzankov A, Li WW, Li VW, Mentzer SJ, Jonigk D (2020). Pulmonary Vascular Endothelialitis, Thrombosis, and Angiogenesis in Covid-19. N Engl J Med..

[122] Dolhnikoff M, Duarte-Neto AN, de Almeida Monteiro RA, da Silva LFF, de Oliveira EP, Saldiva PHN (2020). Pathological evidence of pulmonary thrombotic phenomena in severe COVID-19. J Thromb Haemost.

[123] Cui S, Chen S, Li X, Liu S, Wang F (2020). Prevalence of venous thromboembolism in patients with severe novel coronavirus pneumonia. J Thromb Haemost.

[124] Helms J, Tacquard C, Severac F, Leonard-Lorant I, Ohana M, Delabranche X (2020). High risk of thrombosis in patients with severe SARS-CoV-2 infection: a multicenter prospective cohort study. Intensive Care Med..

[125] Maatman TK, Jalali F, Feizpour C, Douglas A, McGuire SP, Kinnaman G (2020). Routine venous thromboembolism prophylaxis may be inadequate in the hypercoagulable state of severe Coronavirus Disease 2019. Crit Care Med..

[126] Sakr Y, Giovini M, Leone M, Pizzilli G, Kortgen A, Bauer M (2020). Pulmonary embolism in patients with coronavirus disease-2019 (COVID-19) pneumonia: a narrative review. Ann Intensive Care.

[127] Borba MGS, Val FFA, Sampaio VS, Alexandre MAA, Melo GC, Brito M (2020). Effect of high vs low doses of chloroquine diphosphate as adjunctive therapy for patients hospitalized with severe acute respiratory syndrome Coronavirus 2 (SARS-CoV-2) infection: a randomized clinical trial. JAMA Netw Open.

[128] Moores LK, Tritschler T, Brosnahan S, Carrier M, Collen JF, Doerschug K, et al. Prevention, diagnosis and treatment of venous thromboembolism in patients with COVID-19: CHEST guideline and expert panel report. Chest. 2020.

[129] Flaczyk A, Rosovsky RP, Reed CT, Bankhead-Kendall BK, Bittner EA, Chang MG (2020). Comparison of published guidelines for management of coagulopathy and thrombosis in critically ill patients with COVID 19: implications for clinical practice and future investigations. Crit Care..

[130] Robert R, Kentish-Barnes N, Boyer A, Laurent A, Azoulay E, Reignier J (2020). Ethical dilemmas due to the Covid-19 pandemic. Ann Intensive Care.

